# Mild Developmental Foreign Accent Syndrome and Psychiatric Comorbidity: Altered White Matter Integrity in Speech and Emotion Regulation Networks

**DOI:** 10.3389/fnhum.2016.00399

**Published:** 2016-08-09

**Authors:** Marcelo L. Berthier, Núria Roé-Vellvé, Ignacio Moreno-Torres, Carles Falcon, Karl Thurnhofer-Hemsi, José Paredes-Pacheco, María J. Torres-Prioris, Irene De-Torres, Francisco Alfaro, Antonio L. Gutiérrez-Cardo, Miquel Baquero, Rafael Ruiz-Cruces, Guadalupe Dávila

**Affiliations:** ^1^Cognitive Neurology and Aphasia Unit and Cathedra ARPA of Aphasia, Centro de Investigaciones Médico-Sanitarias, Instituto de Investigación Biomédica de Málaga (IBIMA), University of MalagaMalaga, Spain; ^2^Molecular Imaging Unit, Centro de Investigaciones Médico-Sanitarias, University of MalagaMalaga, Spain; ^3^Department of Spanish Language, University of MalagaMalaga, Spain; ^4^Barcelonabeta Brain Research Center, Pasqual Maragall FoundationBarcelona, Spain; ^5^Department of Applied Mathematics, Superior Technical School of Engineering in Informatics, University of MalagaMalaga, Spain; ^6^Department of Psychobiology and Methodology of Behavioural Sciences, Faculty of Psychology, University of MalagaMalaga, Spain; ^7^Unit of Physical Medicine and Rehabilitation, Regional University Hospital, MalagaMalaga, Spain; ^8^Service of Neurology, Hospital Universitari i Politècnic La FeValencia, Spain

**Keywords:** developmental speech disorders, foreign accent, diffusion tensor imaging, personality, psychiatric disorders

## Abstract

Foreign accent syndrome (FAS) is a speech disorder that is defined by the emergence of a peculiar manner of articulation and intonation which is perceived as foreign. In most cases of *acquired* FAS (AFAS) the new accent is secondary to small focal lesions involving components of the bilaterally distributed neural network for speech production. In the past few years FAS has also been described in different psychiatric conditions (conversion disorder, bipolar disorder, and schizophrenia) as well as in developmental disorders (specific language impairment, apraxia of speech). In the present study, two adult males, one with atypical phonetic production and the other one with cluttering, reported having *developmental* FAS (DFAS) since their adolescence. Perceptual analysis by naïve judges could not confirm the presence of foreign accent, possibly due to the mildness of the speech disorder. However, detailed linguistic analysis provided evidence of prosodic and segmental errors previously reported in AFAS cases. Cognitive testing showed reduced communication in activities of daily living and mild deficits related to psychiatric disorders. Psychiatric evaluation revealed long-lasting internalizing disorders (neuroticism, anxiety, obsessive-compulsive disorder, social phobia, depression, alexithymia, hopelessness, and apathy) in both subjects. Diffusion tensor imaging (DTI) data from each subject with DFAS were compared with data from a group of 21 age- and gender-matched healthy control subjects. Diffusion parameters (MD, AD, and RD) in predefined regions of interest showed changes of white matter microstructure in regions previously related with AFAS and psychiatric disorders. In conclusion, the present findings militate against the possibility that these two subjects have FAS of psychogenic origin. Rather, our findings provide evidence that mild DFAS occurring in the context of subtle, yet persistent, developmental speech disorders may be associated with structural brain anomalies. We suggest that the simultaneous involvement of speech and emotion regulation networks might result from disrupted neural organization during development, or compensatory or maladaptive plasticity. Future studies are required to examine whether the interplay between biological trait-like diathesis (shyness, neuroticism) and the stressful experience of living with mild DFAS lead to the development of internalizing psychiatric disorders.

## Introduction

Foreign accent syndrome (FAS) is a stigmatizing disorder that is defined by the emergence of a peculiar manner of articulation and intonation which is perceived as foreign (Whitaker, 1982; Blumstein et al., 1987; Berthier et al., 1991). In the vast majority of individuals with FAS the condition is acquired (AFAS) after brain damage or it emerges during the course of psychiatric illnesses (e.g., schizophrenia). Even less frequently, FAS occurs during speech-language development (Mariën et al., 2009; Keulen et al., 2016a). In these cases, toddlers develop the language (lexicon and grammar), but not the pronunciation (accent) that is peculiar to the community to which they belong (Flege et al., 2006). On exploring this issue in our unit, we were confronted with two different situations; one was that of adolescents with autism spectrum disorder (Asperger’s syndrome) born in families with strong local accents but who spoke with clear standard Spanish accent.^1^ A tentative explanation could be that these children absorb different words from different sources (other people, mass media)^2^ and they use them as “formulaic language” (Locke, 1997) that may be exact replicas of what they had heard during language learning (a way of delayed echolalia for accent) so that their accent may sound as foreign or atypical.

The other situation, which motivates the present study, was the case of two adult males who claimed to have FAS since their early adolescence [developmental FAS (DFAS)]. They realized having DFAS after being alerted by classmates, but they remained uninformed about the nature of the condition until they accessed information on mass media. Both subjects reported on the negative emotional, social, and occupational consequences of speaking with a non-native accent. We selected these two subjects for a more in depth evaluation according to the following criteria: (a) claims by both subjects that their speech sounded foreign to naive listeners since their adolescence (Blumstein et al., 1987); and (b) presence of atypical phonetic features that might explain why their speech was perceived as foreign. This was confirmed by an expert phonetician with experience in FAS (IM-T). On the initial interview, one subject reported family history of stuttering and the other one’s speech resembled cluttering. Moreover, we noticed that they were excessively concerned with their foreign accent; this presumably resulted from the presence of long-standing co-morbid psychiatric conditions (neuroticism, obsessive-compulsive disorder, anxiety, depression, and social phobia) and from limited coping strategies which were heightened by impolite reactions of others. Thus, the presence of psychiatric symptoms raised the possibility that the origin of FAS in these two cases might be psychogenic (Reeves and Norton, 2001; Van Borsel et al., 2005; Verhoeven et al., 2005; Reeves et al., 2007; Keulen et al., 2016b). Nevertheless, changes in brain function and anatomy have been implicated in the pathogenesis of developmental speech disorders including cluttering (Ward et al., 2015) and stuttering (Neef et al., 2015) as well as in AFAS (Fridriksson et al., 2005; Katz et al., 2012; Moreno-Torres et al., 2013; Tomasino et al., 2013). Furthermore, comorbid psychiatric disorders (e.g., obsessive-compulsive disorder, social phobia) can be associated with changes in regions reported to be altered in FAS (Pujol et al., 2004; Fan et al., 2012; Li et al., 2014; see further details below). This means that FAS should not be classified as psychogenic before performing detailed neuroimaging studies.

Since only three cases of DFAS have been reported up to now (Mariën et al., 2009; Keulen et al., 2016a), there is no information about the interaction between the neural systems underpinning speech and behavior in DFAS. Data from a well-studied developmental speech disorder (stuttering) indicates that concomitant disorders (e.g., neuroticism, anxiety, depression, and emotional/behavioral problems) depend on a complex interaction between biological (genetics), psychological vulnerabilities, temperament, cognitive styles, and familiar and peer influences (see Bleek et al., 2012; Alm, 2014; Gunn et al., 2014; Iverach and Rapee, 2014). Therefore, we examined DFAS from a multidimensional perspective, which includes linguistic characteristics, accompanying cognitive and psychiatric deficits, and white matter microstructure with magnetic resonance imaging (MRI) and diffusion tensor imaging (DTI).

The current (limited) knowledge on DFAS cases may be illuminated by prior data on AFAS. Results from previous studies reveals that AFAS is highly heterogeneous from a phonetic viewpoint. Subjects with AFAS may show different patterns of suprasegmental (prosodic) and segmental (consonant and vowel) errors. Suprasegmental errors seem to affect very different aspects (intonation, stress, and emotional prosody), and within one single aspect, error patterns seem to be contradictory. For instance, while some studies reported excessive and atypical pitch contours (Blumstein et al., 1987; Berthier et al., 1991; Ingram et al., 1992; Takayama et al., 1993), others have observed reduced fundamental frequency (F0) ranges (Graff-Radford et al., 1986). Similarly, at the segmental level errors on both vowels and consonants have been observed. Errors in vowels include tensing (Whitaker, 1982; Van Lancker et al., 1983; Blumstein et al., 1987; Ingram et al., 1992), lengthening (Ardila et al., 1988), and schwa coloring (Whitaker, 1982; Gurd et al., 1988). Several studies have documented an overall reduction in the acoustic vowel space due to a restricted F1 range (Ingram et al., 1992; Kurowski et al., 1996; Moreno-Torres et al., 2013). The most commonly cited errors in consonants include manner changes (Ardila et al., 1988; Berthier et al., 1991; Ingram et al., 1992; Moreno-Torres et al., 2013) and voicing errors (Blumstein et al., 1987; Ardila et al., 1988; Gurd et al., 1988), while nasalization (Lippert-Gruener et al., 2005) and place of articulation changes (Whitaker, 1982; Ardila et al., 1988) are less common. Thus, as is the case for prosodic errors, no clear error pattern has emerged consistently in these subjects. The high heterogeneity of these patients explains the difficulty to characterize them, and to determine whether FAS is a syndrome or merely an epiphenomenon (Blumstein and Kurowsky, 2006). However, it has been proposed that the core deficits in these patients might be prosodic, with segmental deficits being secondary (Blumstein and Kurowsky, 2006). According to this proposal, the error patterns might be heterogeneous for three reasons: (1) patients with FAS might differ in the precise prosodic deficit and in its severity; (2) different subtypes of prosodic deficits might produce different patterns of segmental errors, which might vary cross-linguistically; and (3) many patients may have other deficits apart from the prosodic one (e.g., apraxia of speech, dysarthria). This emphasizes the need to examine how different prosodic deficits might disturb speech production. Two situations have been described. One group of patients seems to be characterized by slow articulation and staccato rhythm, which results in frequent consonant strengthening errors and a reduced vowel space (e.g., Ingram et al., 1992; Moreno-Torres et al., 2013). Note that strengthening errors vary cross-linguistically. In the case of Spanish, strengthening processes have been observed with the three voiced stop consonants (/b, d, g/). Typical speakers produce them as approximants [i.e., (β, ð, γ)] (Martínez-Celdrán, 1998). In contrast, FAS patients and many late L2 learners produce them as stops. In another group of patients the speech rhythm is not slow, but they may produce frequent pauses and variable consonant distortions (Gurd et al., 1988). However, not many studies have analyzed the interaction between prosodic and segmental errors. Although the number of subjects with DFAS described until now is scant (Mariën et al., 2009; Keulen et al., 2016a), it remains to be explored whether or not the same linguistic heterogeneity described in AFAS can be observed in developmental cases. It is hypothesized that the results of the present study would provide further information on this issue.

Information on the emotional consequences of AFAS is scarce (Miller et al., 2011; Moreno-Torres et al., 2013) and data reported on DFAS revealed normal behavior (Mariën et al., 2009; Keulen et al., 2016a). Therefore, since the two subjects included in this study complained of cognitive failure and psychiatric symptoms, a second aim our study was to identify the type and severity of these complaints and their potential relationship to FAS. This is a key issue as the nosological status of these psychogenic cases is still controversial (Verhoeven et al., 2005), but note that some guidelines for the diagnosis of the syndrome in clinical practice have recently been made (Keulen et al., 2016b). The term “psychogenic” has always been applied to individuals who show symptoms despite lacking evidence of organic damage (see Vuilleumier, 2005). Nevertheless, recent neuroimaging studies have revealed functional and structural brain changes in archetypal psychogenic disorders such as motor conversion paralysis (Vuilleumier, 2005; Aybek et al., 2014), functional dysphagia (Suntrup et al., 2014), psychogenic amnesia (Botzung et al., 2007), and adult-onset stuttering triggered by stressful life events (Chang et al., 2010). Moreover, cases of FAS have been classified as psychogenic in patients with bipolar disorder and schizophrenia (Reeves and Norton, 2001; Reeves et al., 2007) which have a well-defined neural basis (Vargas et al., 2013; Wheeler and Voineskos, 2014) and even in a patient with mild traumatic brain injury (Cottingham and Boone, 2010). Finally, it is noteworthy that in two cases of AFAS the initial label “psychogenic” was changed to “neurogenic” after demonstrating structural changes (cerebral atrophy, infarcts) and metabolic abnormalities on neuroimaging (Poulin et al., 2007; Moreno-Torres et al., 2013). This is not an inconsequential matter because testimonies from FAS persons reveal that obtaining a proper diagnosis helps to mitigate the negative consequences of speaking with a foreign accent (Miller et al., 2011) preventing dysfunctional adjustment and coping strategies (Moreno-Torres et al., 2013). The question that now arises is which brain regions participate in the co-expression of abnormal speech production and psychopathology in DFAS.

Brain imaging studies on DFAS have revealed normal gross anatomy but decreased activity of several components of the large-scale bilateral speech production network including the cerebellum, basal ganglia, and prefrontal-medial frontal regions (Mariën et al., 2009; Keulen et al., 2016a), a variety of regions which are also involved in cases of AFAS (see Carbary et al., 2000; Scott et al., 2006; Katz et al., 2012; Moreno-Torres et al., 2013; Tomasino et al., 2013, for reviews). Other affected regions in DFAS (left thalamus and lateral temporal regions and occipital cortex bilaterally; Mariën et al., 2009; Keulen et al., 2016a) have also been described in cases of AFAS, yet these areas are not integral components of the speech production network (Wise et al., 1999; Riecker et al., 2005; Sörös et al., 2006; Ackermann and Riecker, 2010). This latter finding increases the likelihood that abnormal activity in such regions could be the consequence of disorders other than DFAS, but which may coexist with it. Therefore, the third aim of the present study was to examine the neural substrate of DFAS and its cognitive and psychiatric comorbidity using DTI to identify changes in white matter microstructure. A comprehensive analysis of the neural substrate underlying developmental speech-language disorders potentially evolving to FAS (e.g., stuttering, specific language impairment) and their comorbid psychiatric disorders [anxiety, social phobia, obsessive compulsive disorder (OCD), neuroticism and so forth] observed in our subjects is beyond the scope of this study (see Servaas et al., 2013; van der Velde et al., 2013; Aghajani et al., 2014; LeWinn et al., 2014; Piras et al., 2015).

## Clinical Case Studies

### Subject 1

Subject 1 was a 27-year-old right handed male who contacted us via e-mail for evaluation of a possible FAS. The family history was unremarkable except for the presence of persistent developmental stuttering in the father. The parents were not related to each other. Developmental history was self-reported and there was no opportunity to obtain information from family members. He was the product of a normal pregnancy and delivery. Developmental milestones were apparently normal and he denied learning disability or specific problems with speech-language, reading and writing, but described himself as a shy child (distress in some social situations; Miskovic and Schmidt, 2012). He grew up in a bilingual environment and his mother tongue (L1) was Spanish. He had learned Valencian (a dialectal variant of Catalan) at home and English at school and in USA where he lived 1 year at the age 9 years. However, since adolescence he did not like to use either Valencian or English in casual conversations because he felt his articulation in these languages was abnormal. Subject 1 attended normal schooling and passed all grades uneventfully. He progressed adequately in high school and college and obtained a degree in Library Science (see further details in psychiatric status and social-occupational adjustment section). Neurological examination was normal showing no motor coordination disorders. He had a digital anomaly (camptodactyly) in several toes but no other developmental malformations.

### Subject 2

Subject 2 was a 46-year-old right handed male who contacted us via e-mail for evaluation of a possible FAS. The family history was unremarkable and his parents were not related to each other. Similarly to what occurred in subject 1, details of subject 2’s developmental history were obtained from his testimony because there was no opportunity to obtain information from family members. He was the product of a normal full-term pregnancy and delivery. Early development was apparently normal but he described himself as a clumsy child with short attention span but no hyperactivity. Subject 2 was normal in language and reading acquisition, but he described occasional stuttering and deficient fine motor skills (e.g., difficulty tying shoe laces) with elements of motor and spatial dysgraphia (inappropriately sized and spaced letters, misspelled words). He grew up in a bilingual environment and his mother tongue (L1) was Spanish. Even though he was born in Catalonia, he had learned Catalan at the age of 14 years when he entered to a Catalan speaking high school. However, he had difficulties mastering Catalan accent to the extent that he disliked speaking this language. As an adult he lived in England and USA during short periods and he reported problems to learn vowels in English, but otherwise his grammar and vocabulary was above average. He attended normal schooling and passed all grades uneventfully. He progressed adequately in high school and college and obtained a Law degree (see further details in psychiatric status and social-occupational adjustment section). Subject 2 commented that he received musical lessons during childhood but he had difficulties to sing even the easiest melodies. Moreover, he reported having problems to impart affective intonation and that his linguistic prosody in everyday communication was abnormal. During adolescence when he wanted to question something he ended the phrase saying “I am asking you.” Neurological examination revealed “soft neurological signs” (Hollander et al., 1990) including impaired finger-to-nose coordination on the right side and right/left confusion. He also had mild buccofacial apraxia.

### Analysis of Foreign Accent

#### Perception

The two subjects and the healthy control subjects signed an informed consent for participation after receiving an explanation of the aims and methodology of the study according to the Declaration of Helsinki. The study protocol was approved by the Ethical Committee of University of Malaga, Malaga, Spain. In order to discard the presence of perception deficits, both subjects were evaluated with a battery of perception tests previously used in our Lab in FAS patients (see Moreno-Torres et al., 2013). This battery includes both segmental (i.e., minimal pairs of words and non-words) and suprasegmental tasks (i.e., lexical stress → /’PA.pa/ vs. /pa.’PA/; intonation → exclamative vs. interrogative). As for both tasks the subjects scored at ceiling, further analysis was focused on productions aspects.

#### Production

Since it was expected that speech production deficits in these subjects were mild it was considered that spontaneous speech samples would be more informative than repetition data. Thus, one 15 min sample of informal conversation was obtained for each subject. The conversation was audiotaped for later analysis using a FOSTEX-LE2 recorder and an Audiotechnica AT2035 microphone. The recording took place in a silent room in our Lab. In addition, to allow for direct comparison with our database from four healthy males, the two subjects were required to produce speech in two conditions: sentence repetition and non-word repetition. The sentence repetition subtest from the Psycholinguistic Assessments of Language Processing in Aphasia (PALPA; Kay et al., 1992; Valle and Cuetos, 1995) was used. This task (PALPA 12) evaluates the ability to repeat auditorily presented sentences (*n* = 36) of different length (from 5 to 9 words). It is composed of reversible sentences (*n* = 20) and non-reversible sentences (*n* = 16). The Aguado’s task was used to explore non-word repetition. This task has a total of 80 tokens divided in two sets of 40, for frequent and infrequent syllables, respectively. The two sub-lists are balanced on the number of non-words of each syllable length (range: 2–5 syllables; Aguado, 2011).

### Analysis of Production Data

#### Segmental Errors

Segmental errors in consonants were grouped as: voicing, nasalisation, place of articulation and manner of articulation. For vowels, errors were grouped as: place of articulation and manner of articulation. Voicing and manner of articulation errors in consonants were further classified as strengthening (fortition processes) or weakening (lenition processes; see Introduction).

#### Suprasegmental Errors

The analysis was based on the sentence repetition task and on the informal conversation. Two aspects were of particular interest, intonation contours and rhythm. Both aspects were analyzed with the help of Praat (Boersma and Weenink, 2010). For intonation contours we obtained measures of F0 range and form by examining the contrast between interrogatives and declarative sentences. In order to explore syllable rhythm, we calculated the speech rate (syllables per second) and its variability (standard deviation).

#### Degree of FAS

Ten students of Speech Pathology rated the degree to which the accent of subject 1, subject 2 and two healthy controls might sound foreign or native. The judges heard a total of four imitated sentences. A Likert scale was used to judge the degree of foreignness, with “1” corresponding to definitely foreign speaker; “2”: probably foreign speaker; “3”: probably native speaker; and “4”: definitely native speaker. Judges were blind to the purpose of the study, other than having to rate items for foreignness Anticipating that due to the mild manifestation of FAS judges might not detect the presence of a foreign accent, another pool of 10 judges were asked to rate the degree of regional accent of the subjects and also of three control subjects. In each case the controls and the judges were from the same region as the subject, Valencian for subject 1 and Catalan for subject 2. For this task, the scores of the Likert scale were “1” corresponding definitely to another dialect; “2”: probably speaker of another dialect; “3”: probably speaker of my dialect; and “4”: definitely speaker of my dialect.

### Cognitive and Language Testing

Handedness was assessed with the Edinburgh Handedness Inventory (Oldfield, 1971) and general intellectual abilities with the Mini Mental State Examination (Folstein et al., 1975). Executive functions were assessed with the Trail-Making Test (parts A and B), the Hayling test (Burgess and Shallice, 1996) and the Stroop Color-Word Test (Stroop, 1935). Verbal attention and memory for word lists were assessed with the Wechsler Memory Scale III (WMS-III; Wechsler, 1997) and visual memory with the Rey-Osterrieth Complex Figure (Rey, 1941; Peña-Casanova et al., 2009). Language functions (phonological, lexical, and semantic) were tested with several subtests of the PALPA (Kay et al., 1992; Valle and Cuetos, 1995) and the Boston Naming Test (short version) (Kaplan et al., 2001). The Controlled Oral Word Association Task (COWAT; Borkowski et al., 1967) and Category Fluency (animal naming) were used to examine phonemic and semantic fluency, respectively (Tombaugh et al., 1999). Communication in activities of daily living was assessed with the Communicative Activity Log (CAL; Pulvermüller and Berthier, 2008). Although the CAL was devised for persons with aphasia, recent data demonstrated that it is also useful to identify decrements in the amount and quality of communication in persons with AFAS (Moreno-Torres et al., 2013). The CAL was completed by the patients and in the case of subject 1 it was also completed by her partner.

### Psychiatric Testing

Since the subjects had a long-lasting history of obsessive-compulsive symptoms, anxiety, and depression, several psychiatric rating scales were administered. Personality and the impact of living with a non-native accent were also examined.

#### Obsessive Compulsive Disorder

The presence of OCD was assessed with the Leyton Obsessional Inventory (LOI; Cooper, 1970) and the Yale-Brown Obsessive Compulsive Scale (Y-BOCS; Goodman et al., 1989). The LOI is a 69-item questionnaire to rate obsessive symptoms (questions 1–46) and traits (questions 47–69). Subscales of the LOI to rate *resistance* to and *interference* of symptoms were not administered and symptom severity was rated with the Y-BOCS. The Y-BOCS is a rating scale intended for use as a semi-structured interview. It rates the obsessions and compulsions and their severity. Scores of symptom severity are as follows: subsyndromal: 0–7; mild: 8–15; moderate: 16–23; severe: 24–31; and extreme: 32–40.

#### Non-OCD Anxiety

Non-OCD anxiety was assessed with the Hamilton Anxiety Scale (HAS; Hamilton, 1959), a 14-item clinician-rated scale that measures psychic anxiety (mental agitation and psychological distress) and somatic anxiety (physical complaints related to anxiety). Each item is scored on a scale of 0 (not present) to 4 (severe), with a total score range of 0–56. Scores < 17 indicate mild severity, 18–24 mild to moderate severity, and 25–30 moderate to severe. Since some patients with FAS tend to avoid social contacts and have reduced functional communication (Miller et al., 2011; Moreno-Torres et al., 2013) the presence and severity of social phobia were assessed with the Social Phobia Inventory (SPIN; Connor et al., 2000). The SPIN is a 17-item self-rating scale that includes items assessing symptom domains of social anxiety disorder (fear, avoidance, and physiologic arousal). The SPIN items are measured by a choice of five answers based on a scale of intensity of social phobia ranging from “not at all” to “extremely.” Overall assessment is done by total score, and a total score higher than 19 indicates a likelihood of social anxiety disorder (scores ranging from 21 to 30 indicate mild severity, 31 to 40 moderate severity, 41 to 50 severe, and 51 or more very severe). Subject 1 also met diagnostic criteria of the Diagnostic and Statistical Manual of Mental Disorders, 5th Edition (DSM-5; American Psychiatric Association [APA], 2013) for a posttraumatic stress disorder (PTSD). These symptoms were evaluated with the 17-item Davidson Trauma Scale (DTS; Davidson et al., 1997), a self-report measure that assesses the 17 DSM-IV symptoms of PTSD. Items are rated on 5-point frequency (0 = “not at all” to 4 = “every day”) and severity scales (0 = “not at all distressing” to 4 = “extremely distressing”). Subject 1 was asked to identify the trauma that was most disturbing to him and to rate, in the past week, how much trouble he had had with each symptom. The DTS yields a frequency score (ranging from 0 to 68), severity score (ranging from 0 to 68), and total score (ranging from 0 to 136).

#### Mood, Motivation, and Emotions

Depression was evaluated with the Hamilton Depression Rating Scale (HDRS), a 17-item interviewer-rated scale that measures psychological and autonomic symptoms of depression (Hamilton, 1959). Scores range from 0 to 52, with higher scores representing greater depressive symptoms. The presence of hopelessness was examined with the Beck Hopelessness Scale (BHS), a 20-item self-report inventory designed to measure three major aspects of hopelessness: (i) feelings about the future, (ii) loss of motivation, and (iii) expectations (Beck et al., 1974). Apathy was measured with the Apathy Scale (AS; Starkstein et al., 1992). The AS consist of 14 items rated on a 4-point scale. The total score is 42 and higher scores indicate more severe apathy. Difficulties in recognizing and verbalizing feelings (alexithymia) was assessed using the self-report 20-item Toronto Alexithymia Scale (TAS-20; Bagby et al., 1994). Scores range from 20 to 100 and scores above 61 are considered abnormal.

#### Personality

Personality was assessed with the Zuckerman–Kuhlman Personality Questionnaire (ZKPQ; Zuckerman, 2002). The ZKPQ assesses basic dimensions of personality or temperament particularly those which describe personality traits with biological-evolutionary roots. The ZKPQ consist of 99 dichotomous (true-false response) questionnaire distributed in five content scales, namely Neuroticism-Anxiety (19 items), Activity (17 items), Sociability (17 items), Impulsive Sensation Seeking (19 items), and Aggression-Hostility (17 items) and an Infrequent Scale which is used as a validity index. The psychometric properties in a large sample of Spanish subjects demonstrated that the ZKPQ is a valid self-report measure of personality traits (Gomà-I-Freixanet et al., 2008).

#### Personal Experience of Living with Changes in Accent

The personal experience of living with a change in accent was explored in both subjects using an unstructured interview which was mainly based on their testimonies. Three main topics were addressed during the interview (see Miller et al., 2011). These included: (i) details of accent change (initiation, type of accent) and related problems with prosody and communication; (ii) adjustment and copying strategies to accent; and (iii) reactions of family members and others to accent.

## Neuroimaging

Magnetic resonance imaging studies were performed in the subjects with DFAS and in a group of 21 healthy control right-handed males. Healthy controls were matched with DFAS subjects by gender (all controls were male), age (mean age ± SD: 33.05 ± 10.03 years; range: 22–59 years) and education (controls: from college to university degree). Healthy controls were Spanish speaking male residents in Malaga with variable knowledge of other languages (English and French).

### Image Acquisition

The MRI studies were performed on a 3-T MRI scanner (Philips Gyroscan Intera, Best, The Netherlands) equipped with an eight-channel Philips SENSE head coil. Head movements were minimized using head pads and a forehead strap. High-resolution T-1 structural images of the whole brain were acquired with three dimensional (3D) magnetization prepared rapid acquisition gradient echo (3 D MPRAGE) sequence (acquisition matrix: 240/256; field of view: 240 mm; repetition time (TR): 9.9 ms; echo time (TE): 4.6 ms; flip angle: 8°; turbo field echo (TFE) factor: 200; 0.8 mm × 0.8 mm × 0.8 mm resolution). One hundred eighty two contiguous slices, 0 mm slice gap, were acquired. The total acquisition time of the sequence was about 4:24 min. In addition to the 3D MPRAGE, a standard axial T-2 weighted/FLAIR [TR = 11.000 ms; TE = 125/27 ms; 264 matrix × 512 matrix; field of view (FOV) = 230 mm × 230 mm; 3-mm-thick slices with 1 mm slice gap] was obtained. DTI data acquisition was performed using multi-slice single-shot spin-echo echo-planar imaging (EPI) with specific parameters as follows: FOV 224 mm, 2-mm-thick slices with 0 mm slice gap, TE = 117 ms, TR = 12408 ms, and b factor: 3000 s/mm^2^. The EPI echo train length consisted of 59 actual echoes reconstructed in a 112 × 128 image matrix. Thirty-two diffusion directions were used, and the acquisition was repeated twice in order to enhance the signal to noise ratio.

### Image Processing

Motion and eddy current correction were performed on the DTI images of the two subjects and the 21 healthy controls using the eddy current correction tool (Smith, 2004; Woolrich et al., 2009) within FMRIB’s Diffusion toolbox (FDT) of FSL^3^. The BET tool was used to delete non-brain tissue from the images, and diffusion tensor estimation was carried out using the DTIFIT tool within FDT, with the least-square estimation algorithm. Maps of fractional anisotropy (FA), mean diffusivity (MD), axial diffusivity (AD), and radial diffusivity (RD) were then obtained.

The T1 scans were AC-PC oriented, segmented into gray matter, white matter and cerebro-spinal fluid with the New Segment tool within SPM12. Then the Diffeomorphic Anatomical Registration Through Exponentiated Lie Algebra (DARTEL; Ashburner, 2007; Bergouignan et al., 2009; Klein et al., 2009) toolbox was applied to the segmented tissue maps in order to register them to the stereotactic space of the Montreal Neurological Institute (MNI). The B0 maps were used for a rigid coregistration of the DWI maps to the T1 AC-PC oriented images. This was carried out with the FLIRT tool from the FSL toolbox. Then a non-rigid coregistration of the FA images to the space of the white matter native segment was performed with the FNIRT toolbox. The same transformation was applied to the other DWI maps. This was done in order to remove distortions of the DWI images and adapt them to the T1 scan before normalization. The DARTEL tissue deformations were then used to normalize the participants’ FA, MD, AD, and RD maps to the MNI space. Finally, the resulting FA, MD, AD, and RD maps were written with an isotropic voxel resolution of 1.5 mm × 1.5 mm × 1.5 mm and smoothed with a 12 mm full with half maximum (FWHM) Gaussian kernel.

### Statistics

A voxel-based analysis on the FA, MD, AD, and RD maps was performed with SPM12. Given that each of the two subjects could present with specific abnormalities, each of them was separately compared to the group of healthy controls in a two sample *t*-test. Moreover, to study if there were effects shared by the two subjects with DFAS, a 2-sample *t*-test was also performed between the group of the two subjects and the control group. A set of brain regions was pre-selected to perform voxel-based comparisons with small-volume correction (SVC). Regions-of-interest (ROIs) were selected from TD Labels, WFU PickAtlas Tool. Specific ROIs previously described to be involved in AFAS (Katz et al., 2012; Moreno-Torres et al., 2013; Tomasino et al., 2013) and in associated psychiatric disorders (OCD, social phobia, depression, neuroticism, and alexithymia; Servaas et al., 2013; van der Velde et al., 2013; Aghajani et al., 2014; Piras et al., 2015) were selected. The following regions were included for analysis: bilateral orbital cortex, left inferior frontal gyrus, left precentral gyrus, left insula, left inferior parietal lobe, left posterior cingulate, left inferior temporal gyrus, left orbital cortex, left anterior cingulate, left cingulate gyrus, left superior frontal gyrus, left caudate, left lentiform nucleus, left middle frontal gyrus, and right middle frontal gyrus. In this analysis, voxels were regarded as significant when falling below a corrected voxel threshold of.05 (family wise error – FWE) adjusted for the small volume. In an initial analysis, differences were also assessed at the whole-brain level, in order to find any significant effects taking place in other areas. Voxels of *p* < 0.005 (uncorrected, cluster size ≥ 100 voxels) were considered as indicating significant difference between subjects and the control group. Both SVC and whole-brain analyses were also carried out for four randomly selected control subjects to rule out false positives. In the whole brain analysis clusters above the selected level were found for control subjects in various cases; therefore whole brain results were not considered significant for the patients.

## Results

### Linguistic Data

#### Subject 1

**FIGURE 1:**
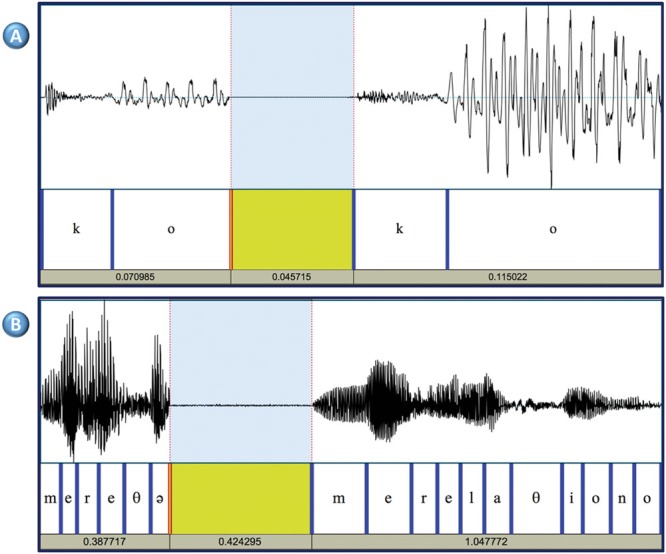
**(A)** Consonant strengthening (or vowel weakening) Syllable /ko/ produced by subject 1 (left) and a control subject (right). The amplitude of the signal in the VOT interval is almost identical in the two subjects (46–47 dB), but the vowel is produced with more energy by the control subject (peak intensity: 52 dB in subject 1, and 67 dB in the control subject). **(B)** Example of cluttered speech in subject 2. The left part shows one unintelligible fragment produced by subject 2 when attempting to produce the words “me relaciono” (“I interact”). The right part corresponds to the standard production by a typical speaker. These two words were part of a long utterance (“Pues es complicado conocer gente allí; pues me relaciono con gente en general” (“So, it is not easy meeting people there. I interact with different people”). Subject 2 omits five out of eleven phonemes and distorts one vowel. The resulting sequence is non-intelligible and out of context.

Results revealed that linguistically and phonologically subject 1 could be considered a typical speaker. In the three speech perception tasks he scored at ceiling (words minimal pairs: 20/20; non-words minimal pairs: 19/20; lexical stress: 10/10; intonation: 20/20). As anticipated due to the mildness of FAS, subject 1 also scored at ceiling in the sentence imitation task (35/36) and in the non-word imitation task (77/80). Detailed linguistic analyses indicated that the production of subject 1 was phonologically very close to typical. At the prosodic level, there was no evidence of atypical production in any of the tasks: subject 1 produced the typical F0 contours (e.g., descending intonation for declaratives, ascending intonation for interrogatives), and the F0 range could be considered as typical (e.g., neither flat nor excessively variable). At the segmental level, subject 1 did produce some errors, however, they represented less than 3% in the non-word repetition task, and less than 1% in the spontaneous speech sample. Altogether, this suggests that subject 1 did not have relevant phonological difficulties. In contrast, phonetically his production was not fully typical, as observed particularly in the frequent production of consonant strengthening. The phenomenon was relatively frequent with the / b / phoneme (19/58), and less frequent with the / d / (4/21). Strengthening was not observed with the / g /. Fortitions were specially frequent in specific phonetic contexts: in / lb / sequences (9/9), as in “alberto” (Albert); in /rb/ sequences as in “árbol” (tree), (2 /7); in /sd/ sequences as in “desde” (from; 3/5); in /𝜃d/ as in “en vez de” (instead of; 1/1). Acoustic analyses provided further evidence of atypical phonetic characteristics of subject 1. In typical Spanish speakers there is a clear contrast in terms of amplitude or intensity between the VOT of a voiceless stop and the following vowel, whereas in subject 1 the two sounds had similar amplitudes (**Figure 1A**). This might indicate that subject 1 was reinforcing the consonant, or that he was weakening vowels. In order to measure this phenomenon the intensity contrast of 20 /kV/ sequences in a VkV context; e.g., /k/ and /o/ in the sequence /pako/) was calculated. The same measure was obtained from four typical speakers. The results revealed that the mean contrast in the controls was 18.6 dB (Range 15.5 dB: 21.0 dB) whereas in subject 1 the contrast was 11.1 dB. An independent samples *t*-test confirmed that the difference was significant (*p* < 0.01) between subject 1 and each of the controls. Seven judges considered that subject 1 was definitely a native speaker and the remaining three expressed some doubts and could only state that subject 1 was possibly a native speaker. Accordingly, the mean score was 3.7. This score was not significantly different for the one obtained by the controls (range: 3.8–4). A difficulty emerged with the evaluation of the dialectal origin by the naïve judges. The judges did not agree on the dialectal origin of the controls (i.e., some controls were classified as non-local). A close examination of the results revealed that the judges’ ratings might be influenced by the fact that in Valencia there are two official languages (Valencian and Spanish). Some judges classified as Valencian only those control subjects whose Spanish showed a strong influence of Valencian (e.g., with a strong /l/) (Briz, 2004). For the rest of the controls, whose first language was Spanish, and also for subject 1, the judges disagreed, which indicates that regional accent of subject 1 was not easily distinguishable from that of the control subjects.

#### Subject 2

Similarly to the other subject in this study, subject 2 scored at ceiling in various tasks. In the perception tasks the scores were 20/20 (words minimal pairs), 18/20 (non-words minimal pairs), 9/10 (lexical stress), and 20/20 (intonation). In the sentence imitation task the score was 36/36. However, the results of the non-word and the spontaneous speech sample indicated that his production was not fully typical, both at the suprasegmental and at the segmental levels. At the suprasegmental level, preliminary analyses indicated that while the speech rate and rhythm in the repetition tasks was normal, in the spontaneous sample the speech rate was atypically high, which seemed to reduce intelligibility. Also, the speech rate in the non-word and the sentence repetition tests was almost identical to the speech rate of one control subject [(mean syllables/second ± SD] subject 2: 4.6 ± 8; control subject: 4.7 ± 9]. In contrast, the rate in the speech sample was atypically high (8.7 ± 1.9 syllables/second). Data from the four healthy control subjects showed that the mean was 7.1 ± 2.1 syllables/second. This suggests that while in the imitation tasks, subject 2 tended to use the same speech rate as the model, in free speech he accelerated his emissions. Segmental errors were observed in all the conditions. In the non-word repetition test, subject 2 produced incorrectly 11% of the consonants (i.e., these errors were four times more frequent than in subject 1). Errors were varied, including consonant distortions and substitutions, and syllable or word structure errors. Consonant errors included nasalizations (*n* = 2), de-nasalization (*n* = 1), place or articulation (*n* = 2), manner or articulation (*n* = 2), and l > ɾ (*n* = 2). Syllable/word structure errors included vowel insertion (*n* = 2), consonant insertion (*n* = 1), consonant omission (*n* = 1), and metathesis (*n* = 3). Errors were also varied in the other two conditions (i.e., sentence imitation and spontaneous speech). However, in the spontaneous speech sample segmental errors were more severe, included diverse suprasegmental errors. For instance, 3% of the words were completely unintelligible. Further, subject 2 tended to omit consonants (>4%), syllables (>2%) and some grammatical words (e.g., determiners). An illustrative example of accelerated production in subject 2 is shown (**Figure 1B**). Altogether, these results indicate that the difficulties in speech production were clearly higher in the spontaneous speech samples than in the imitation tests. The mean score in the accent perception task was 3.5. This result indicates that the five judges (50%) were unsure about the origin of subject 2’s accent. This score was not significantly different from that of the controls. Such result is compatible with the data presented above showing that while the speech of subject 2 was mostly typical, he did produce some errors which may have raised the doubts of the judges. The exploration of the dialectal origin of subject 2 raised the same problems that had been observed with subject 1 (i.e., the judges classified as dialectal only those controls who spoke Spanish with a very strong Catalan accent). And the judges disagreed as to the origin of subject 2 and the other controls.

### Cognitive Findings

Results from cognitive and language testing of both subjects are shown in **Table 1**. Both subjects were right-handed and they did not show general cognitive deficits. Both subjects had normal language production in the sense that they did not show word finding difficulties or grammatical and syntactic anomalies. Nevertheless, subject 2 had some disruption in the flow of verbal messages (fast rate of speaking, excessive collapsing, or deletion of syllables; see above) indicative of cluttering (St. Louis and Schulte, 2011). On subtests of PALPA, both subjects obtained normal scores in subtests tapping phonology, lexical and semantic processing, although subject 2 had a slightly decreased performance on auditory lexical decision of non-words. Their performance on semantic and phonological fluency were normal. Executive functions were normal, but subject 2 showed impaired performance on part B of the Sentence Completion Hayling Test showing greater response latencies and difficulty inhibiting incorrect words. Scores on verbal learning and memory for word lists ranged from low average to well-above average, whereas visual memory was moderately impaired in both subjects, a finding already reported in subjects with OCD (Shin et al., 2014). Communication in activities of daily living (CAL) was decreased in both subjects, particularly in subject 1 most likely due to social phobia. Further item-by-item analysis of the CAL revealed that subject 1 had major problems in communicating with foreigners, with several others he does not know, in offices, stores or public institutions, under stress or when he was tired. Also, he rarely verbally respond to criticisms. Self-reflections on communication skills measured with CAL in subject 1 were more negative than the ones reported by his partner (a 26 year-old female) on the same scale (subject 1: frequency: 42; partner: frequency: 65, *p* = 0.0009, Fisher Exact Test, two-tailed; subject 1: quality: 53; partner; quality: 68 *p* = 0.0266, Fisher Exact Test, two-tailed). Scores on the CAL were also abnormal in subject 2 particularly in making statements or reports about facts, using the telephone, answering questions asked by others, under stress, or when he was tired.

**Table 1:** Cognitive testing.

	Subject 1		Subject 2	
Edinburgh Handedness Inventory (laterality quotient, LQ)^∗^	+90	Right handed	+85	Right handed
Mini Mental State Examination	30	Normal	30	Normal
Psycholinguistic Assessments of Language Processing in Aphasia^∗∗^				
Non-word minimal pairs (*n* = 56)	53	Normal	54	Normal
Word minimal pairs (*n* = 56)	56	Normal	55	Normal
Auditory lexical decision				
Word (*n* = 80)	79	Normal	80	Normal
Non-word (*n* = 80)	78	Normal	74	Abnormal
Repetition, syllable length (*n* = 24)	24	Normal	23	Normal
Repetition: non-words (*n* = 24)	22	Normal	22	Normal
Spoken word-picture matching (*n* = 40)	40	Normal	39	Normal
Auditory sentence comprehension (*n* = 60)	**–**	Not tested	56	Normal
Auditory sentence comprehension of locative relations (*n* = 24)	–	Not tested	22	Normal
Digit production	7	Normal	7	Normal
Boston Naming Test (short form, 15-items)	14	Normal	15	Normal
Controlled Oral Word Association Task (FAS)	42	Normal (44.7 ± 11.2^†^)	43	Normal (44.7 ± 11.2^†^)
Semantic fluency (animal naming)	22	Normal (21.9 ± 5.4^†^)	23	Normal (21.9 ± 5.4^†^)
Communicative Activity Log				
Frequency (maximum = 90)	42	Impaired	70	Mildly impaired
Quality (maximum = 90)	53	Impaired	70	Mildly impaired
Trail Making Test				
Part A (seconds)	26	Normal (24.40 ± 8.7^§^)	34	Normal (31.78 ± 9.93^§^)
Part B (seconds)	50	Normal (50.68 ± 12.36^§^)	59	Normal (63.76 ± 14.42^§^)
Test de Hayling				
Part A				
Latency (seconds)	17.5	Normal (12.6 ± 6.5^ƪ^)	19.95	Normal (12.6 ± 6.5^ƪ^)
Part B				
Latency (seconds)	22	Normal (26.4 ± 19.5^ƪ^)	64.1	Abnormal (26.4 ± 19.5^ƪ^)
B-A latencies	4.5	Normal (13.8 ± 16.3^ƪ^)	44.15	Abnormal (13.8 ± 16.3^ƪ^)
Error score (unrelated, %)	80%	Normal (83.3%^ƪ^)	20%	Abnormal (83.3 %^ƪ^)
Stroop color-word test (*T* scores)				
Color	42	Normal	50	Normal
Word	44	Normal	40	Normal
Color-word	56	Normal	58	Normal
Interference	62	Normal	62	Normal
Wechsler Memory Scale-III: Word list (scaled scored)				
First recall (attention)	9	Average	10	Average
Total recall	8	Average	6	Low average
Rate of learning	14	Well above average	8	Average
Short-term recall	5	Well below average	10	Average
Long-term recall	7	Low average	9	Average
Recognition	10	Average	8	Average
Percentage of retention	6	Low average	10	Average
Rey–Osterrieth complex figure		Percentil range^¶^		Percentil range^¶^
Copy	36	>99	32	41–59
Delayed reproduction (40 min)	12.5	19–28	9	11–18

*^∗^Caplan and Mendoza, 2011; ^∗∗^Valle and Cuetos, 1995; ^†^Normative data from Tombaugh et al., 1999; ^§^ Normative data from Tombaugh, 2004; ^ƪ^Normative data from Burgess and Shallice, 1996; ^¶^Peña-Casanova et al., 2009.*

### Psychiatric Status

Both subjects fulfilled DSM-5 (American Psychiatric Association [APA], 2013) criteria for OCD, generalized anxiety disorder, depression, and for other psychiatric conditions (**Table 2**). The two subjects obtained abnormal scores on obsessional traits (LOI-traits items) and also reported multiple obsessions and compulsions (LOI-symptoms items). On both the LOI and Y-BOCS symptom checklist they described aggressive and contamination obsessions as well as compulsions including checking, repeating, and hoarding rituals (subject 1) and cleaning/washing, checking, repeating, counting, hoarding (subject 2). Subject 1 also described perfectionism and pathological doubting, whereas subject 2 reported “need to know” obsessions and engaged in making excessive lists and mental play. Both subjects had generalized anxiety disorder without panic attacks or agoraphobia and subject 1 additionally met diagnostic criteria for social phobia, PTSD, and alexithymia. Subject 2 had “possible” alexithymia and although he described discomfort in some social situations, he did not meet criteria for social phobia. Both patients also had depression, hopelessness, and apathy. Assessment of personality with the ZKPQ revealed increased scores in the Neuroticism-Anxiety factor in both subjects and decreased scores in the Sociability factor in subject 1. None of them met diagnostic criteria for hypochondriasis or other somatoform disorders.

**Table 2:** Psychiatric testing.

	Subject 1		Subject 2		
Leyton Obsessional Inventory^∗^				
Symptoms	19		26	
Traits	12		13	
Total	31	Symptomatic	39	Symptomatic
Yale-Brown Obsessive Compulsive Scale				
Obsessions	10		11	
Compulsions	6		8	
Total	16	Moderate severity	19	Moderate severity
Hamilton Anxiety Scale				
Psychic anxiety	14		13	
Somatic anxiety	12		18	
Total	26	Clinically significant^†^	31	Clinically significant^†^
Social Phobia Inventory	36	Clinically significant^¶^	14	Normal
Davidson Trauma Scale				
Frequency	29		–	
Severity	24		–	
Total	53	Clinically significant	–	
17-item Hamilton Depression Rating Scale	22	Moderate depression	23	Moderate depression
Beck Hopelessness Scale	11	Moderate severity	14	Moderate severity
Apathy Scale	7	Mild severity	15	Moderate severity
Toronto Alexithymia Scale	81	Alexithymia^§^	55	Possible alexithymia^§^
Zuckerman–Kuhlman Personality Questionnaire		Normal scores		Normal scores
Neuroticism-Anxiety	16^ƪ^	6.74 ± 4.41	l4^ƪ^	6.74 ± 4.41
Activity	11	8.53 ± 3.50	9	8.53 ± 3.50
Sociability	2^‡^	7.02 ± 3.49	7	7.02 ± 3.49
Impulsive Sensation Seeking	6	8.03 ± 4.27	9	8.03 ± 4.27
Aggression-Hostility	6	6.95 ± 3.26	9	6.95 ± 3.26

*^∗^The LOI was used to assess the presence of obsessive and compulsive symptoms, whereas the Y-BOCS was additionally used to rate symptom severity; ^†^scores > 14 in the Hamilton Anxiety Scale are clinically significant (Bech, 1993); ^¶^ a cut-off score of 19 distinguishes well between clinical populations of phobia patients (Connor et al., 2000); ^§^ scores ≥ 61 = alexithymia, scores between 52 and 60 = possible alexithymia (Bagby et al., 1994); ^ƪ^indicates that both patients were 1 standard deviation above the mean (Gomà-I-Freixanet et al., 2008) whereas ^‡^indicates 1 standard deviation below the mean (Gomà-I-Freixanet et al., 2008).*

### Social-Occupational Adjustment

Regarding the personal experience of living with FAS both subjects reported that after taking notice on its existence in the media, they sought to confirm their provisional self-diagnosis of FAS. On interviews to assess their personal experience of living with FAS, both subjects stated that they realized for the first time that they spoke with foreign accent during adolescence when classmates commented about their extraneous manner of intonation. It was noteworthy that no one among their close relatives was aware of accent change in patients 1 and 2. The presence of persistent developmental stuttering in the father of subject 1, might explain why mild speech production problems in subject 1 passed undetected to his relatives. Subject 1 commented that entering high school was a terrifying and an overwhelming experience because his new classmates repeatedly asked him if he was a foreigner due to his manner of speaking. Most classmates (and later on other people) considered that his accent variously resembled French, American English, Argentinean, or Mexican Spanish, Rumanian, and Italian, while others simply contended that his accent sounded foreign. One adverse consequence of accent change in subject 1 what that his classmates used to call him “the foreigner.” He was also deeply ashamed and unhappy by the fact that he was not recognized as a citizen of Valencia. He abstained from speaking in social situations to the extent that he remained silent when he met somebody on the street. By that time, subject 1 developed intrusive and avoidant symptoms (PTSD) and he became obsessed with his foreign accent. Moreover, when somebody asked him about either his origin or manner of speaking he ruminated about these questions during several days. He described no close friends and reported that his atypical accent and social anxiety made difficult for him to establish new social relationships. In the case of subject 2, he also realized that his speech sounded foreign when he was alerted by his classmates at high school. Through several years naïve listeners (classmates and coworkers) considered that his accent variously resembled Spanish from Lerida (a region in the west Catalonia), South-America, or the Canary Islands, or French, while others simply contended that his accent sounded foreign or uncommon. The most negative consequence of his accent was that it impeded him from obtaining a qualified job as attorney or commercial worker.

Both subjects believed that the deviation of their accents from the native prototype hampered the possibility of obtaining a job commensurate with their qualifications. In addition, subject 2 reported that in one occasion while living in Madrid he failed obtaining a job as a teacher of Spanish for foreigners in a telephone interview because of his distinct accent. Subject 1 had worked at home as a freelance website designer because he had problems to obtain other types of jobs. Both subjects had adjustment problems related to the consequences of living with FAS, yet the negative impact was more pronounced in subject 1. On the SPIN (an scale to assess social anxiety), subject 1 obtained the higher scores on questions related to verbal communication (“I avoid talking to people I don’t know,” “fear of embarrassment causes me to avoid speaking to people,” “talking to strangers scares me,” and “I avoid having to give speeches”) in part because he disliked his accent and tone of voice. As already stated, he also reported symptoms of increased psychological sensitivity and arousal as well as avoidant behavior consistent with PTSD. On answering the question of the DTS which enquires about the precipitant event(s) for developing PTSD he considered that the only traumatic event which lead to PTSD was the consequence of living with FAS.

## Neuroimaging Findings

### Structural MRI

**FIGURE 2:**
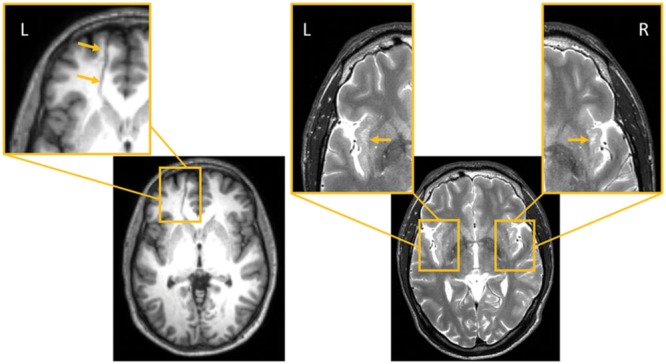
**(Left)**: Subject 1. (1) Axial FLAIR MRI image shows a transmedullary vein close to the head of the left caudate nucleus crossing the medial frontal lobe substance and reaching the superior longitudinal sinus. This region has been expanded to depict the venous angioma (orange arrows; left panel, inset). **(Right)**: Subject 2. (2) Axial T_2_-weighted axial MRI imaging shows enlarged perivascular spaces in both insular cortices; the largest one are in the left anterior insula (orange arrows, insets). L indicates left and R right sides of the brain.

Magnetic resonance imaging in subject 1 showed a venous angioma close to the head of the left caudate that crossed the medial frontal lobe white matter to drain into the superior sagittal sinus (Lee et al., 1996; Osborn et al., 2004; **Figure 2**). There also was a mild dilatation of the left lateral ventricle, but the rest of the MRI did not show other structural brain anomalies. In subject 2, the MRI showed expanded perivascular spaces (EPVS) mainly involving both insular cortices (Song et al., 2000; **Figure 2**).

### Diffusion Tensor Imaging

**FIGURE 3:**
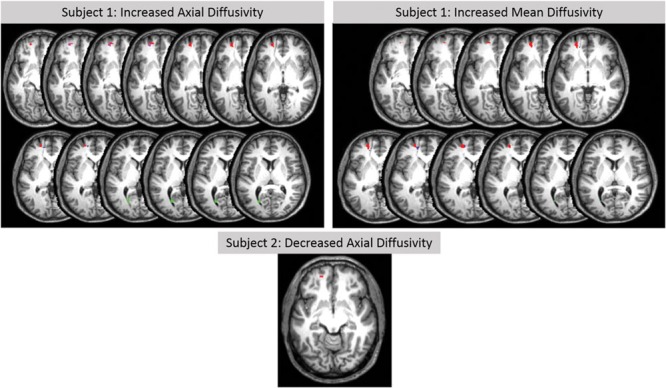
**T_1_-weighted axial MRI images of DTI-based analysis in subjects 1 and 2.** Increases in AD (left top images) and in MD (right top images) are shown in subject 1 in the left superior frontal gyrus (red), left middle frontal gyrus (magenta), left anterior cingulate gyrus (blue), and left posterior cingulate gyrus (green). A decrease in AD (medial bottom image) is shown in subject 2 in the left superior frontal gyrus (red; decreased MA in the same ROI is not shown). Significant changes (small volume correction, *p* < 0.05 corrected) are relative to a healthy control subjects.

Although changes of white matter microstructure were found in both subjects, comparison of the two subjects together with a group of 21 age- and gender-matched healthy controls revealed no differences. Individual analyses of DTI-based microstructural changes showed significant changes in both subjects in some predefined ROIs of the left hemisphere in comparison to the healthy control group. No changes were found in the right hemisphere with this methodology. **Table 3** shows MNI coordinates and cluster sizes of the different diffusion parameters in Subject 1; these parameters for subject 2 are shown below. Voxel-based comparisons with SVC (*p* < 0.05, FWE corrected) in subject 1 showed significant increases values of MD, AD, and RD in regions surrounding the venous angioma and affecting the superior frontal gyrus, medial frontal gyrus and anterior cingulate gyrus. Increased AD and MD were also identified in subject in the posterior cingulate gyrus (**Figure 3**). In subject 2, small clusters of decreased MD (superior frontal gyrus, seven voxels, *x* = -20, *y* = 52, *z* = -10) and AD (superior frontal gyrus, nine voxels, *x* = -18, *y* = 54, *z* = 10) were found in the left frontal lobe (**Figure 3**). Analysis of four healthy control subjects revealed only false positive results in one of them in two clusters. One cluster of 12 voxels was found in the left inferior temporal gyrus (*x* = 45, *y* = -66, *z* = -2) and another cluster of two voxels was found in the left superior frontal gyrus (*x* = -22, *y* = 4, *z* = 57).

**Table 3:** Areas of abnormal DTI-derived parameters in subject 1 compared to 21 healthy control subjects.

	Region	p FWE-corrected^∗^	*t*-value	Peak coordinates^∗∗^	Cluster size
				*x y z*	
MD+	WM_L_Anterior_cingulate gyrus	0.008	4,54	-18 50 0	15
	WM_L_Anterior_cingulate gyrus	0.048	3,58	-20 42 -2	1
	WM_L_Posterior_cingulate gyrus	0.042	3,41	-26 -66 4	6
	WM_L_Superior_frontal_gyrus	0.003	5,39	-21 52 -4	135
AD+	WM_L_Anterior_cingulate gyrus	0.034	3,75	-18 50 0	7
	WM_L_Middle_frontal_gyrus	0.004	5,37	-21 54 -6	37
	WM_L_Posterior_cingulate gyrus	0.014	3,97	-22 -69 4	55
	WM_L_Superior_frontal_gyrus	0.004	5,3	-21 54 -4	103
RD+	WM_L_Anterior_cingulate gyrus	0.014	4,23	-18 50 0	6
	WM_L_Middle_frontal_gyrus	0.014	4,77	-21 52 -6	17
	WM_L_Middle_frontal_gyrus	0.043	4,18	-24 48 -2	2
	WM_L_Superior_frontal_gyrus	0.006	5,06	-21 52 -3	110

*^∗^FEW indicates family wise error; ^∗∗^peak coordinates represent the location of the maximum pixel values in standard Montreal Neurological Institute (MNI) space. MD+ indicates elevated mean diffusivity, AD+ elevated axial diffusivity, and RD+ elevated radial diffusivity. WM indicates white matter and L, left side of the brain.*

## Discussion

In the present study, we have described the case of two adult subjects presenting with mild DFAS. Speech development was presumably normal in subject 1, yet his father had persistent developmental stuttering. Subject 2 also had a positive history for stuttering and now he shows cluttering. Our findings suggest that DFAS can occur in the context of different speech and language deficits (Mariën et al., 2009), a finding previously reported in AFAS (Coleman and Gurd, 2006; Katz et al., 2012; Moreno-Torres et al., 2013). Although both subjects (1 and 2) had reported that naïve listeners (classmates, friends, and acquaintances) frequently perceived their speech as foreign, naïve judges did not confirm this possibility. Given these results it could be argued that these two subjects should not be described as FAS cases. However, detailed phonetic analyses revealed that both subjects produced errors that might explain that under certain circumstances they were perceived as foreigners. Subject 1 produced frequently consonant strengthening errors. These errors consist in producing clearly a sound that is typically weakened. Consonant weakening is very selective and it is constrained by rhythmic factors. For this reason, whenever a speaker cannot use the local rhythm, he or she strengthens sounds that are normally weak. Typically, this happens in foreign speakers and in FAS cases, both of which tend to articulate too slowly for weakening processes to occur. In other words, the subtle phonetic errors observed in subject 1 might be caused by an underlying suprasegmental deficit (i.e., rhythmic), similar to the ones observed in other FAS cases (Ingram et al., 1992).

As for subject 2, our results indicated that his speech was also rhythmically atypical, with a tendency to accelerate excessively, resulting in an increased percentage of consonant errors particularly omissions, and even unintelligible production. Such speech disorder is suggestive of a mild form of cluttering (St. Louis and Schulte, 2011). The current working definition of cluttering (St. Louis et al., 2007) is a fluency disorder characterized by a speech rate that is perceived to be abnormally rapid, irregular or both. These rate abnormalities further may be result in one or more of the following features: (1) excessive disfluencies; (2) abnormal pauses, syllable stress, or speech rhythm; and (3) inappropriate degrees of co-articulation among sounds, especially in multisyllabic words. This means that while both FAS and cluttering are prosodic deficits that cause segment impairments, cluttering is characterized by atypical speech rhythm whereas FAS is characterized by slow rhythm and possibly atypical intonation patterns (Blumstein and Kurowsky, 2006; Moreno-Torres et al., 2013). In light of the syndromic overlap it is not surprising that the speech production of subject 2 was perceived as foreign by many listeners. Also note that these rhythmic errors were not observed in imitation, which suggests that when subject 2 has a model he can regulate the rhythm of his emissions. However, he might not be able to regulate it by himself during narrative.

Altogether, this suggests that there are coincidences and differences between subjects 1 and 2. The clearest coincidence is that in both subjects the underlying linguistic deficit seems to be related with prosody, and particularly with rhythm, a deficit that results in varied segmental errors. This result would support the proposal that the core deficit in some FAS cases is a prosodic one (Blumstein et al., 1987). The difference between the two subjects is that the prosodic deficits are qualitatively different. While subject 1 tended to articulate slowly in all task conditions (i.e., imitation/spontaneous speech), which might be related with a difficulty to plan/execute phonological programs (Moreno-Torres et al., 2013), subject 2 instead tended to articulate too rapidly exclusively in spontaneous speech, a pattern reflecting his inability to regulate the rhythm.

### Cognitive Deficits and Psychiatric Comorbidity

Comprehensive testing of cognitive functions in previous DFAS cases yielded mixed results (see details in Mariën et al., 2009; Keulen et al., 2016a). One such patient had discrepant performance (better verbal than non-verbal scores) on intelligence and memory tests scoring in the lower range on visual search and sequencing (Patient TL, Mariën et al., 2009). Another patient had abnormal performance on abstract concept formation, set shifting, and maintaining goal-oriented strategies as well as abnormal visual memory with low average scores on visual-motor integration and coordination (Keulen et al., 2016a). However, the remaining patient had normal cognitive functioning (Patient KL, Mariën et al., 2009). Analysis of these data indicate not only that non-linguistic cognitive deficits are not a prerequisite for the occurrence of DFAS, but also that the detection of such deficits may point to the dysfunction of other neural networks or of a single network subserving more than one function. Cognitive testing in our subjects also revealed deficits beyond the speech-language domain, yet we did find fewer and milder impairments than in previous DFAS cases (Mariën et al., 2009; Keulen et al., 2016a). Although further studies are required to investigate the nature of such deficits, we suggest that the pattern of impairment in our subjects might be related to comorbid psychiatric disorders rather than being central constituents of DFAS. Indeed, the most consistent and severe deficit in our subjects was on visual memory (delayed reproduction of the Rey–Osterrieth Complex Figure), an alteration frequently reported among individuals with OCD (Shin et al., 2014). In addition, subject 2, who has had attentional deficits during childhood, demonstrated poor inhibition of automatic responses in part B of the Hayling Sentence Completion Test. Changes in communication have been described in previous FAS cases (Miller et al., 2011; Moreno-Torres et al., 2013) and our subjects were not the exception. However, in the present cases communication deficits may be directly linked to DFAS, at least in subject 2. Both subjects had decreased communication but the origins of such deficits may be different in subject 1 as his scores on the CAL were lowered mainly due to increased anxiety and avoidance in social situations involving speech (specific social phobia; Stein and Deutsch, 2003), a combination previously reported among individuals with stuttering (Iverach and Rapee, 2014).

Mood and behavior in previous cases of DFAS were not affected (Mariën et al., 2009; Keulen et al., 2016a). By contrast, our subjects had psychiatric complaints, a warning sign that prompted a detailed psychiatric evaluation. The liaison between psychological factors (e.g., personality traits, anxiety, and stress) and developmental speech-language disorders has been repeatedly mentioned in the literature (Snowling et al., 2006; Dietrich et al., 2012; Gunn et al., 2014; Karukivi and Saarijärvi, 2014) and it involves a complex and multifaceted cross-talk. For example, a central question is whether psychological symptoms once established persist in individuals with developmental speech-language disorders or if they actually develop in latter phases as aftermaths of delayed communication (Alm, 2014; Iverach and Rapee, 2014). In the case of DFAS, note that even if accent changes emerge during early childhood, the emotional impact of this way of speaking may not be apparent until adolescence when the use of language for social adjustment is more demanding (Mariën et al., 2009). Furthermore, studies of children with specific language impairment have shown that this diagnosis during childhood has some relations to adult psychosocial outcomes (Snowling et al., 2006; Whitehouse et al., 2009; Karukivi et al., 2012). Data from the two subjects described herein illuminates up to what point living with a mild form of DFAS may have consequences for social, emotional and occupational adjustment. Psychiatric examination in subjects 1 and 2 revealed a variety of disorders, specifically internalizing disorders including OCD, non-OCD anxiety, social phobia (subject 1), depression, apathy, and personality features of obsessive-compulsive disorder, neuroticism-anxiety, reduced sociability (subject 1), and alexithymia. This constellation of psychiatric disorders did not conform to previous reports of psychogenic FAS (Reeves and Norton, 2001; Verhoeven et al., 2005; Poulin et al., 2007; Reeves et al., 2007). Rather, associated psychopathology to DFAS in our subjects was remarkably similar to that reported by children and adults with stuttering and cluttering (Alm, 2014; Gunn et al., 2014; Iverach and Rapee, 2014). This was not totally unexpected in our cases as subject 1 had a positive family history of persistent developmental stuttering in his father and subject 2 had stuttering during childhood and now shows cluttering.

### Gross Structural Brain Anomalies: Incidental or Symptomatic?

In the present study, structural brain anomalies were identified on the MRI of both subjects, a venous angioma in the left frontal lobe in subject 1 and EPVS in both insular regions in subject 2. Some radiological studies and textbook descriptions considered that these gross anomalies are incidental MRI findings lacking clinical relevance (Song et al., 2000; Osborn et al., 2004). Nonetheless, venous angiomas involving the frontal lobe can be symptomatic and present with psychiatric symptoms even in the absence of hemorrhagic complications (Nagaratnam et al., 1990; Watanabe et al., 2005). Cerebral venous angiomas are presumably secondary to a primary dysplasia of capillaries and small transcerebral veins or represent a compensatory mechanism caused by an intrauterine occlusion of a normal venous system (Lee et al., 1996). Parenchymal changes adjacent to the venous angioma have been described (Santucci et al., 2008) and correspond to demyelination, gliosis, leukomalacia, and neuronal degeneration (Noran, 1945) or result from compensatory adaptive changes secondary to hemodynamic disturbances (Kimura and Mitake, 2001; Watanabe et al., 2005; Santucci et al., 2008). Our findings suggest that the anatomical distribution of the venous angioma in subject 1 may have altered intrinsic connectivity within the frontal lobe as is probably reflected by significant increases of MD, AD, and RD in regions (left superior frontal gyrus, anterior cingulate gyrus) close to the venous angioma. In the case of subject 2 structural MRI revealed multiple confluent EPVS involving anterior and posterior insular regions bilaterally. The nature and clinical significance of EPVS is still under debate (Dávila et al., 2010) and it has been suggested that EPVS involving the insular cortex lack clinical relevance (Song et al., 2000). Nevertheless, this subject had mild DFAS, poor motor coordination, and difficulties in sequencing of complex motor tasks (mild buccofacial apraxia), all abnormal features previously linked to insular involvement (Tognola and Vignolo, 1980; Moreno-Torres et al., 2013). Thus, our findings align with results from previous studies linking EPVS with developmental disorders including autistic disorder (Boddaert et al., 2009), Tourette’s syndrome (Dávila et al., 2010), and coordination disorder (Brockmann et al., 2009).

### Abnormal White Matter Microstructure

Note that visual inspection of structural MRI in all three cases of DFAS reported so far failed to found structural brain anomalies (Mariën et al., 2009; Keulen et al., 2016a) indicating that the responsible pathological substrate is below the resolution of the naked eye and pass undetected unless more sophisticated neuroimaging methods (e.g., DTI voxel-based morphometry, positron emission tomography) are used (Moreno-Torres et al., 2013). In fact functional imaging in a case of DFAS disclosed decreased perfusion in several regions (Mariën et al., 2009) including areas (bilateral prefrontal cortex, medial frontal regions, and cerebellum - Keulen et al., 2016a) of the large-scale bilateral speech production network (Wise et al., 1999; Riecker et al., 2005; Guenther et al., 2006; Sörös et al., 2006; Eickhoff et al., 2009; Ackermann and Riecker, 2010; Bohland et al., 2010). Our study is the first one that used DTI to examine microstructural changes in DFAS. Comparison of our two subjects together with the group of age- and gender-matched healthy controls revealed no differences in DTI parameters. However, individual DTI-based analysis restricted to predefined ROIs revealed subtle abnormalities. Subject 1 did show elevated MD, AD, and RD values relative to controls in key components of speech and emotion regulation networks. Altered DTI-based parameters were found in the left hemisphere involving the superior frontal gyrus, the middle frontal gyrus, and the anterior and posterior cingulate cortex. These regions have been reported to be involved in AFAS (see Moreno-Torres et al., 2013) and in previous neuroimaging studies of the psychiatric disorders presented by our subjects (e.g., OCD, social phobia, alexithymia, neuroticism; Pujol et al., 2004; Fan et al., 2012; Servaas et al., 2013; Karukivi and Saarijärvi, 2014). In subject 2, small clusters of decreased MD and AD were found in the left superior frontal gyrus. Thus, the left superior frontal gyrus and its surrounding regions most likely played a role in clinical symptoms in both subjects as well as in another case of DFAS (Keulen et al., 2016a). Nevertheless, MD, AD, and RD were increased in subject 1, whereas MD and AD 2 were decreased in subject. This might have resulted because altered white matter integrity around the venous angioma in subject 1 could have a different pathological substrate than in subject 2. Changes in diffusion parameters in both subjects may variously result from disrupted neural organization during development, maladaptive neural plasticity resulting from a cognitive and emotional bias toward negative emotions (Koganemaru et al., 2012), or inhibition of emotional expression due to heightened reappraisal (Giuliani et al., 2011) triggered by the negative and stressful consequences of having speech production deficits (Dietrich et al., 2012).

Our findings also revealed that co-occurring mild FAS and developmental speech disorders (atypical phonetic production, cluttering) may be associated with abnormal emotion regulation. The involvement of some regions (insula and cingulate gyrus) related to the emergence of foreign accent are also important for the expression personality traits (neuroticism and alexithymia) and psychiatric disorders (OCD, anxiety, social phobia, PTSD, depression, apathy, and hopelessness) diagnosed in our subjects (Pujol et al., 2004; Servaas et al., 2013; van der Velde et al., 2013; Aghajani et al., 2014; LeWinn et al., 2014; Piras et al., 2015). Involvement of these regions and also of frontal lobe regions (middle frontal gyrus) important for motor control of voice and speech production might explain in our subjects the dysfunctional interaction between personality traits, response to stress, and speech production (Dietrich et al., 2012). Thus, an altered interplay between biological trait-like diathesis (shyness and neuroticism) and the stressful experience of living with DFAS might explain the development of internalizing psychiatric disorders during late adolescence.

## Author Contributions

All authors listed, have made substantial, direct and intellectual contribution to the work, and approved it for publication. MLB, NRV, IMT, and GD were involved in conception and design, acquisition of data, or analysis and interpretation of data. MLB, IMT, GD, MJTP, and IDT performed language, cognitive and behavioral evaluations. NRV, CF, KTH, RRC, FA, and JPP interpreted neuroimaging data. MBL, NRV, IMT, and GD drafted the article and revised it critically for important intellectual content.

## Conflict of Interest Statement

The authors declare that the research was conducted in the absence of any commercial or financial relationships that could be construed as a potential conflict of interest.

## Footnotes

1 Specifically, in the area where these children live, the phoneme / s / is produced systematically as an interdental fricative [ 𝜃 ], a phenomenon which is known as *ceceo*. Ceceo is socially stigmatizing and unacceptable in most of the country. When examining children from areas in which *ceceo* is common, we found that the phenomenon was observable in typical children (i.e., they produced / s / as [ 𝜃 ]) but not in those with Asperger syndrome (i.e., they produced / s / as [ s ], as in standard Spanish; Moreno-Torres, I., and Rodríguez-Ibanéz, D., unpublished).

2 http://www.wrongplanet.net/forums/viewtopic.php?t=184538

3 http://www.fmrib.ox.ac.uk/fsl/
